# Epigenetic Age Acceleration and Chronological Age: Associations With Cognitive Performance in Daily Life

**DOI:** 10.1093/geroni/igaf122.2055

**Published:** 2025-12-31

**Authors:** Stacey Scott, Daisy Zavala

**Affiliations:** Stony Brook University, Stony Brook, New York, United States; Florida State University College of Medicine, Tallahassee, Florida, United States

## Abstract

DNA methylation-derived epigenetic clocks offer the opportunity to examine aspects of age acceleration, which differ among individuals and may better account for age-related changes in cognition. Leveraging existing ambulatory cognitive assessments in daily life from a sample of 142 middle-aged adults, we examined associations between measures of epigenetic age acceleration and performance on tasks of processing speed and working memory. Covarying for chronological age, we used multilevel models to examine associations of epigenetic age acceleration with both average level and variability of cognitive performance. Positive age acceleration was associated with poorer mean processing speed and working memory. Greater chronological age was associated with poorer mean processing speed and working memory performance. Further, positive age acceleration was associated with greater intraindividual variability, and greater chronological age was associated with less. Our results suggest that epigenetic age acceleration may account for additional risk and interindividual variation in cognitive performance above chronological age.

